# “Just realising that I wasn’t alone… was profound”: a mixed-methods evaluation of a pilot peer-to-peer wellbeing program for carers of children with rare epilepsies

**DOI:** 10.1186/s13023-025-04036-0

**Published:** 2025-10-21

**Authors:** Kristine Pierce, Jack Bernard Murphy, Eden G. Robertson, Jahidur Rahman Khan, Susan Bullock, Cristol Barrett O’Loughlin, Megan Loden, Rebecca McIntosh, Erin Beavis, Natalie Roberts, Elizabeth Emma Palmer, Raghu Lingam

**Affiliations:** 1 Discipline of Paediatrics and Child Health, School of Clinical Medicine, Faculty of Medicine and Health, UNSW Sydney, Sydney, NSW Australia; 2 Child UnLimited, Randwick, NSW Australia; 3 Raregivers Inc, Orange, CA 92869 USA; 4 Sydney Children’s Hospitals Network, Randwick, NSW Australia

**Keywords:** Rare disease, Carers, Strengths-based, Wellness, Wellbeing, Rare epilepsy, Social connection, Developmental and epileptic encephalopathy

## Abstract

**Background:**

Caring for a child with a complex rare condition is widely acknowledged to have a significant impact on mental health and wellbeing. However, carers often report a lack of appropriate evidence- and strengths-based resources and services that can pre-emptively support their wellbeing. Rare epilepsies are a group of highly complex, typically childhood-onset conditions, characterised by unpredictable seizures, multi-morbidities, lack of targeted treatments, and reduced life expectancy and quality of life. We evaluated the acceptability, feasibility and early effectiveness of a 6-week online group wellness program for Australian carers of a child with rare epilepsy. We co-designed the evaluation with a multidisciplinary team with lived experience and expertise in consumer engagement, medicine, psychology, and implementation science.

**Methods:**

The acceptability, feasibility and impact of the program on carers' self-reported wellbeing, self-efficacy and social inclusion was evaluated using a mixed-methods pilot study. The primary outcomes of acceptability and feasibility were assessed post-intervention using a survey measure with 15 purpose-designed items. Secondary outcomes including participant wellbeing, quality of life, and social inclusion were assessed using validated measures pre- and post-intervention. Semi-structured interviews with retreat participants explored in more depth the study’s perceived strengths, limitations and value. Recordings were transcribed verbatim and analysed for emergent themes.

**Results:**

Ten participants completed both the pre- and post-intervention questionnaires, with six of these participants also completing an interview. The retreat was considered highly acceptable and feasible; nine out of ten participants rated the quality of the retreat as ‘Excellent’ and agreed that the sessions were relevant to their experience. There was provisional evidence of early effectiveness, with improvements in wellbeing, self-efficacy, and social inclusion over the course of the study. Qualitative interviews revealed that participants highly valued the retreat’s flexible online delivery and practical nature, and the facilitator’s authentic knowledge of the carer role.

**Conclusion:**

This pilot study supports the potential benefits of this flexible, online program on carer wellbeing and social inclusion. Larger controlled evaluations are needed to further investigate the program’s impact over the short, medium and long-term, including potential enhancements to accommodate more diverse carers and in different settings**.**

**Supplementary Information:**

The online version contains supplementary material available at 10.1186/s13023-025-04036-0.

## Background

Rare diseases encompass over 8000 distinct conditions [1]. The limited availability of clinical expertise and targeted therapies for rare diseases pose significant challenges to affected individuals and their carers [2]. Children with rare epilepsies, such as developmental and epileptic encephalopathies (DEEs), often experience chronic health issues, intellectual disability and/or autism, and are at a high risk of premature death [3]. These conditions necessitate intensive long-term care, placing substantial emotional, physical, and financial burdens on carers [4].

Carers of children with a rare epilepsy syndrome frequently face isolation, heightened stress, and mental health challenges, with studies indicating an elevated risk of depression and anxiety and chronic traumatic stress related to the unpredictable nature of their child’s conditions and risk of progressive symptoms and premature death [5–7]. This mental strain not only affects carers' wellbeing but also the health outcomes of children under their care [8]. While the emotional and physical demands of caring for a child with a rare epilepsy are significant, recent research also highlights potential positive adaptations for caregivers [4]. Robertson et al. (2024) reported that, alongside high stress and disrupted quality of life in parents of children with DEEs, many caregivers described strengthened meaning and purpose, personal growth, and a reframing of life priorities. Consistent with broader evidence, caregiving can, for some families, deepen bonds, enhance emotional insight, and catalyse resilience—even as substantial burden persists [4]. At the same time, reviews note that positive dimensions of caregiving in DEEs remain comparatively under-explored relative to burden-focused work [9].

Australia’s recently published National Recommendations for Rare Disease Health Care highlights the importance of proactive enquiry by clinicians into the mental health and wellbeing of people living with rare diseases and their carers and provision of appropriate support. The mental health impacts of caring for a person with a rare disease are well recognized [10–12], yet there has been little research into the best way to proactively support families, who often struggle to find accessible and appropriate mainstream mental health and wellbeing programs [13]. To date, few intervention programs have been developed and evaluated in either an Australian or global setting [14].

In response to this need and gap, Raregivers Inc (previously Angel Aid), a United States-based nonprofit organisation, developed a 6-week Wellness Retreat program designed to enhance coping skills, improve mental and emotional health, and connect carers to a broader support network. Preliminary evaluations conducted by the Raregivers team (unpublished data) suggest this program is valuable in supporting carers of children with rare conditions, but comprehensive external evaluations are lacking.

This pilot study adapted the Raregivers Wellness Retreat for carers of children with a rare epilepsy in Australia. This mixed methods pilot study aimed to assess the program’s feasibility, acceptability, and preliminary effectiveness of the adapted program in the Australian setting. Specifically, our study sought to answer the following research questions:

1. How acceptable is the Raregivers Wellness Retreat to participating carers?
2. How feasible is the Raregivers Wellness Retreat to participating carers?
3. How does participating in the Raregivers Wellness Retreat impact outcomes including carers' self-reported wellbeing, quality of life, and social inclusion?

## Methods

This study was approved by the University of NSW (UNSW) Sydney Human Research and Ethics Committee: number HC220624.

### Study design

#### Intervention components and adaptation

The Raregivers Wellness Retreat programme was initially designed by Raregivers Inc to support family members caring for a loved one impacted by a rare disease. It included three virtual sessions per week delivered over 6 weeks, focusing on guided exercises, sensory self-care experiences, and the *Raregivers Emotional Journey Map*. The program also incorporated Sustainable Carer Workshops, providing practical strategies for self-care and resilience. Participants engaged in group-based sessions via Zoom™, ensuring flexibility and accessibility. Each session was designed to last approximately 60 min, with additional resources and exercises provided for participants to complete at their own pace throughout the week. The program was free to participants, and each carer received a wellness kit containing educational and self-care materials to enhance their experience.

Adaptation of the program for the Australian context was led by consumer lead (KP) and Raregivers course coordinator (ML), who have lived experience caring for children with complex rare epilepsy syndromes. They co-designed the weekly program to ensure its relevance and acceptability for Australian participants. While the core structure of the program remained consistent with the original design, the adaptation integrated culturally and contextually relevant topics. The co-design process ensured that the program addressed the unique challenges faced by carers in Australia, enhancing its impact and resonance with participants. This iterative approach allowed sessions to evolve in response to participant needs, ensuring the program was both dynamic and participant-centred.

#### Participants

Carers were eligible to participate in the study if they had attended the retreat and met the following criteria: (i) were the primary carer of a child with a complex, rare epilepsy; (ii) were aged 18 years or older; (iii) were able to communicate in English; and (iv) had access to the internet and a computer. Carers were screened for psychological distress via the online expression of interest form, which incorporated a screening questionnaire with a validated Distress Thermometer (see Additional file 1) [14]. Because this was a pilot study, the Wellness Retreat was scoped to establish safety, feasibility, and acceptability. Individuals with high distress scores (≥ 9 on the Distress Thermometer) were therefore not included, as the program was designed as a strengths-based, preventative wellbeing initiative and was not equipped to provide acute psychological support. Eligible participants provided informed consent and were enrolled. Those screening with high distress were individually contacted by the research team, provided information on appropriate mental health services, and offered support to connect with these services if required.

Consent to participate in evaluation of the retreat was given electronically, and implied consent was received from participants who completed and submitted the baseline and follow-up questionnaires. Participants also gave verbal consent prior to starting the interviews.

#### Recruitment

Participants were recruited via the mailing lists and social media platforms (Twitter, Facebook, LinkedIn) of relevant not-for-profit and community organisations, including Genetic Epilepsy Team Australia (GETA), Epilepsy Foundation Australia, Rare Voices Australia, Genetic Alliance Australia, and Syndromes Without A Name Australia. These organisations shared the study advertisement, which included a link to the expression of interest form. Eligible carers received an email invitation to participate, which included a link to the study information sheet and consent form. A maximum of two follow-up attempts (email or telephone call, using contact details provided in the expression of interest form) were made to non-responders.

#### Evaluation design

The acceptability, feasibility and impact of the Raregivers Wellness Retreat on carers' self-reported wellbeing and social inclusion was evaluated using a mixed-methods pilot study. Participants completed a pre-intervention (upon enrolment, before their first session; see Additional file 2) and post-intervention (1 week after the final session; see Additional file 3) questionnaire, administered online via the Research Electronic Data Capture (REDCap) platform [15, 16]. Questionnaires were anticipated to take approximately 30 min to complete.

Following the program, participants were invited to one-on-one semi-structured interviews via Zoom™. These interviews were audio recorded, de-identified, and transcribed for analysis (see Additional file 4 for the interview guide).

#### Measures

The primary outcomes of acceptability and feasibility were assessed post-intervention using 15 purpose-designed items (see Additional file 3).

Secondary outcomes related to participant wellbeing, quality of life, social inclusion and the extent to which participants live consistently with their values (valuing) were assessed using validated scales at pre- and post-intervention (Table 1 and Additional files 2 and 3).

**Table 1:** Overview of carer questionnaire measures/items

Validated measure	Outcome	Items	Response scale
Positive emotion, Engagement, Relationships, Meaning, and Accomplishment (PERMA) Profiler Questionnaire [17]	Wellbeing	15 items	10-point slider scale ranging from 0 (never/terrible/not at all) to 10 (always/excellent/completely), with 0 indicating the lowest wellbeing score, and 10 indicating the highest wellbeing score
Social Inclusion Scale [18]	Social inclusion	17 items with three subscales: Social Isolation, Social Relations and Social Acceptance (2 items in more than one subscale)	4-point Likert scale: 1 (not at all), 2 (not particularly), 3 (yes, a bit), 4 (yes, definitely)
Valuing Questionnaire [21]	Valued life	10 items with two subscales: Progress and Obstruction	Slider scale ranging from 0 (not true at all) to 10 (completely true)
Adult Social Care Outcomes Toolkit (ASCOT-SCT4) [22]	Carer quality of life	7 items	4-level scale ranging from 1 (ideal state) to 4 (high needs)

#### Wellbeing

The 15-item PERMA Profiler questionnaire was utilised to assess wellbeing across five domains [17]. The full PERMA Profiler contains 23 items, however a shortened version with 15 items was used for this study.

#### Social inclusion

The Social Inclusion Scale (SIS) assesses individuals’ self-perceived levels of social inclusion [18–20]. The overall SIS score was computed by summing all responses (which range from 16 to 64). This scale formulates three elements negatively and scores them in reverse order when calculating the overall score.

#### Values

The Valuing Questionnaire was used to assess the extent to which participants lived consistently with their values [21]. The Progress subscale evaluates personal adherence to values, and the Obstruction subscale measures the extent of barriers hindering the pursuit of a valued life. The ratings for each subscale were aggregated.

#### Carer quality of life

The ASCOT-SCT4 [22] was used to assess carer experience and social care-related quality of life across 7 domains: (1) Occupation; (2) Control over daily life; (3) Self-care; (4) Personal safety; (5) Social participation; (6) Space and time to be yourself; (7) Feeling supported and encouraged.

#### Post-intervention interviews

At the conclusion of the program, participants were invited to participate in a one-on-one semi-structured interview that covered their overall impressions of the program, what they liked/disliked, challenges, feasibility and acceptability of the program as well as their wellbeing, self-efficacy and social connectedness. The interview guide (see Additional file 4) was developed by the multidisciplinary team which included an epilepsy nurse, genetic counsellor, psychologist, clinical geneticist, consumer engagement expert and implementation science experts.

#### Quantitative analyses

Data was subjected to descriptive analysis by a statistician independent to the implementation team. Various descriptive statistics were computed, including count, mean, standard deviation, proportion, median, and range. We conducted a comparative analysis of various indicators at two time points, baseline and post-intervention, to examine the changes. Inferential testing for effect were not undertaken due to the small sample size.

For the PERMA profiler [17], the domain specific change was calculated by subtracting participants’ baseline scores from their follow-up scores for each domain. A positive change score indicates improved wellbeing over time, while a negative change score indicates reduced wellbeing over time. We report this data as frequencies of change.

The SIS domain-specific change was computed by subtracting baseline scores from follow-up scores for each domain. Positive change scores suggest greater and stronger sense of social inclusion over time, while negative change scores indicate weaker and lower inclusion.

The domain-specific change for Valuing was calculated by subtracting baseline scores from follow-up scores in each domain. A positive change score in the Progress domain indicates a higher degree of valued living over time, whereas a higher score in Obstruction reflects a lower level of valued living.

For each domain of the ASCOT-SCT4, we compared the pre- and post-intervention scores for each participant. A positive change was defined as one or more category shifts towards an ‘ideal state’, and a negative shift as one or more category shifts towards ‘high level needs’ from baseline to follow-up.

#### Qualitative analysis

The responses to open ended questions and interviews were analysed using a thematic analysis approach to explore the common experiences of participating in the program and highlight the impact of the program, as well as any barriers and facilitators to implementation. All transcripts were coded and analysed in NVivo© Version 12. The analysis followed five key stages outlined by Braun et al. (2019) [23]: data familiarisation, generation of codes, searching for themes, grouping and reviewing themes, and defining and naming themes. The transcripts were first independently read and coded by two researchers (KP, JM). These initial codes were then reviewed and updated following a discussion with the research team (KP, JM, SB). After the initial coding process, themes were generated and discussed with the research team (KP, JM, EEP), including the relationships between themes, and revision and collapsing of overlapping themes.

#### Mixed methods analysis

The quantitative and qualitative data were integrated narratively at the interpretation and reporting level using a weaving approach [24]. The quantitative and qualitative findings are presented together on a theme-by-theme basis according to the outcomes.

## Results

40 carers completed the expression of interest form, 33 of whom were eligible to participate in the program. The remaining 7 were not eligible to participate because their distress level was 9 or greater. Of the 20 people who joined the program and completed at least one session, 15 carers consented to the evaluation and completed the baseline questionnaire, and 10 carers also completed the follow-up questionnaire. In addition, 6 participants (all of whom completed the baseline and follow-up surveys) participated in an interview. Participant demographics are provided in Table 2. The ten participants who completed both baseline and follow-up questionnaires were included in the final analysis. Attendance by participants in the sessions varied (median 7.7 sessions; range 5–11 of 18), reflecting caregiving and scheduling constraints. We inspected attendance as a potential explanatory factor for changes in secondary outcomes, but given the small sample and variability, no clear pattern was evident. [34]

**Table 2:** Socio-demographics of carers

Carer demographics	Carers completing Q1 (n = 15)	Carers completing Q1 and Q2 (n = 10)	Carers completing Q1 and Q2 and interview (n = 6)
Carer age (years) at baseline survey, mean (SD)	41.47 (6.47)	40.23 (5.24)	42.02 (4.53)
Highest completed education, n (%)			
Certificate/diploma	4 (27)	2 (20)	1 (17)
University degree	6 (40)	5 (50)	3 (50)
Postgraduate degree	5 (33)	3 (30)	2 (33)
Employment, n (%)			
Full-time	5 (33)	2 (20)	1 (17)
Part-time	7 (47)	6 (60)	3 (50)
Unemployed	3 (20)	2 (20)	2 (33)
Remoteness^1^, n (%)			
Regional or remote	2 (13)	1 (10)	0 (0)
Major city	13 (87)	9 (90)	6 (100)
Residential area SES quintile^2^, n (%)			
Q1 (Most disadvantaged)	1 (7)	1 (10)	0
Q2	1 (7)	1 (10)	0
Q3	1 (7)	1 (10)	1 (17)
Q4	6 (40)	3 (30)	3 (50)
Q5 (Least disadvantaged)	6 (40)	4 (40)	2 (33)
Other characteristics			
Age of child at baseline survey, mean (SD), years	7.95 (5.23)	6.86 (4.89)	6.84 (4.67)
Average number of days a month spent at doctor’s appointment or in the hospital for child’s condition			
Mean (SD)	3.07 (2.66)	3.20 (2.97)	2.83 (3.76)
Median (range)	2.0 (0–10)	2.5 (0–10)	1.0 (0–10)

*SD* standard deviation, *SES* socioeconomic status; Q1, baseline questionnaire; Q2, follow-up questionnaire. All participants were female and married

^1^Residential remoteness is based on the 2016 Accessibility/Remoteness Index of Australia Plus (ARIA +) classification developed by the Australian Centre for Housing Research and used by the Australian Bureau of Statistics

^2^Residential postcode level Socio-Economic Indexes for Areas (SEIFA) Index of Relative Socio-Economic Disadvantage (IRSD) quintiles based on the 2021 Australian Household and Population Census developed by the Australian Bureau of Statistics [35]. Quintiles were calculated by re-grouping deciles into five classes

The primary and secondary outcomes are outlined below, along with an in-depth qualitative thematic analysis of the major areas that carers chose to share. Results are grouped into three main areas: acceptability and feasibility (primary outcomes); feedback on program structure; and carer wellbeing and social inclusion (secondary outcomes). Each of these areas are expanded and triangulated with qualitative findings.

## Acceptability and feasibility

Overall, carers were satisfied with the Raregivers Wellness Retreat (Table 3), with highly positive scores for the quality, length, online format and relevance of the sessions. Although overwhelmingly satisfied, half of participants reported they had only ‘somewhat’ incorporated self-care and wellness practices into their own time and routines (Table 3).

**Table 3:** Acceptability and feasibility of the Raregivers Wellness Retreat

Question	Response	Number of carers (n = 10)
How would you rate the quality of the service you received with the Wellness Retreat?	Excellent	9
	Good	1
	Fair	0
	Poor	0
To what extent has the Wellness Retreat met your needs?	Almost all of my needs have been met	3
	Most of my needs have been met	6
	Only a few of my needs have been met	1
	None of my needs have been met	0
The program length should have been…	Less than 6 weeks	1
	It was the right length	6
	More than 6 weeks	3
The workshop sessions were…	Too short	1
	They were the right length	9
	Too long	0
The online format of the retreat suited me	Strongly agree	7
	Somewhat agree	3
	Somewhat disagree	0
	Strongly disagree	0
The Sustainable Carer workshops were relevant to my experience	Strongly agree	9
	Somewhat agree	1
	Somewhat disagree	0
	Strongly disagree	0
The workshops on Sensory Self-Care were relevant to my experience	Strongly agree	10
	Somewhat agree	0
	Somewhat disagree	0
	Strongly disagree	0
I enjoyed/found it beneficial having other people in the group	Strongly agree	10
	Somewhat agree	0
	Somewhat disagree	0
	Strongly disagree	0
As a result of the Wellness Retreat, I have incorporated self-care and wellness practices into my own time and routines	Yes	5
	Somewhat	5
	No	0
If a friend were in need of similar help, would you recommend the Carer Wellness Retreat to them?	Yes, definitely	10
	Yes, generally	0
	No, not really	0
	No, definitely not	0
I had enough time and energy to complete the activities and workshops	Strongly agree	3
	Somewhat agree	5
	Somewhat disagree	2
	Strongly disagree	0
Did you like the help you were getting?	Yes	10
	Somewhat	0
	No	0
Have the services helped you with your life?	Yes	9
	Somewhat	1
	No	0

The acceptability and feasibility of the program was explored in more detail using semi-structured interviews. Overall, carers reported that the study was acceptable as it provided a safe space, offered flexibility and gave them the ability to self-direct, and that it was feasible with regards to length, timing and online format.

The retreat brought together parents and facilitators with varied experiences of rare epilepsy to foster a safe environment for parents to share and reflect alongside those with a shared understanding of rare epilepsy. Interviewees found it particularly beneficial to share their experiences with other carers of children with rare epilepsies. *“Hearing that other people had gone through the same things was extremely refreshing”* [Carer 12]. Those at various stages on their rare epilepsy journeys repeatedly noted the value of engaging with others who had had different but related experiences. Those who were earlier on in their journeys took comfort from the more experienced carers *“I was questioning myself… So it was really incredibly powerful to meet other mums who are further along (the journey)”* [Carer 9]. Parents who had been caring for children with rare epilepsy for years had a chance to reflect: “*Having families who are mums on different stages of their journey, and different ideas on how they dealt with things, it also gives that further connection to what I can do to improve my child’s health or my own health*” [Carer 15].

## Theme: “easy to do” skills that “really worked”

All interviewees expressed their approval of the content of the retreat and valued the simplicity and efficacy of the taught techniques. They felt that the practical instruction and experiential learning of the techniques included in the retreat resonated with them more than written instructions would have. “*They were new skills, like the alternate nostril breathing. There were just certain things about it because I've been told to do that before but the way that they did it, she had you put your fingers on your forehead and I don't know there were just bits about it that were in practice with someone instead of just reading a theory about oh, yeah, you should do these and yeah, it's to be able to practice that experiential learning of like, actually doing it and then them actually teaching you properly how to do things*” [Carer 11].

There were mixed responses on whether the skills taught from the retreat were able to be fully incorporated into ongoing daily life. *“You'd find yourself doing the little bits and pieces of those exercises that the therapists told us about different parts of the day. And you're like, oh, yeah, that really worked. Well. Let me do it again. And I still do it. I did it last night before I fell asleep*” [Carer 15]. Shorter skills that could be fitted into busy schedules were particularly valued, for example breathing, self-massage techniques, and use of scented oils “*There was a clear framework … with going through the senses.. using the.. essential oils …for a quick micro reset*” [Carer 7].

However, the participants also appreciated that there was a realistic understanding from the facilitators that the skills may not be able to be consistently applied, aligning with the facilitators’ authentic understanding of the challenges of the rare caregiving role. *“I think, with [a different retreat], there was so much emphasis on doing each of the activities for such a long time. And consistently, it's not really manageable as a carer, whereas this one was like, it's okay if you miss. It felt a bit more supportive*” [Carer 12].

## Theme: “the fact that the people running it understood it from the epilepsy angle was everything”

Participants reported that they found the online retreat proved a safe and nonjudgemental environment for parents to share their experiences with rare epilepsy. “*It was the space for everyone to share…there were a couple of times that it was difficult for me to share. And I didn't feel judged, you know, I just, I felt like that was really okay… the facilitators didn't push. And I don't think that the other participants, you know, felt disappointed or annoyed that I couldn't share, you know, it felt like, yeah, just that it was okay. It was, you know, it felt like it was a safe environment to share if you could, and if you couldn't, that was just as acceptable*” [Carer 12].

The experience of the facilitators with rare epilepsy, and their sensitivity to the challenges of parents, was especially valued. “*You have to have the right people to do it. So the content itself isn't like groundbreaking, you know, we all know meditation and breathing and stuff like that. But the fact that these people running it understood it from the epilepsy angle was everything. So I think it's what, you know, yeah. The content needs the right people delivering it as well*” [Carer 9].

## Theme: online delivery “meant that those rare people could connect”

The online nature of the retreat was overall seen as a suitable delivery format, and indeed that it facilitated the bringing together of parents of children with rare epilepsy who otherwise may not have had the opportunity to meet or connect. “*I don't think it would have been that group of people if it wasn't online. You can really only have a local group if it's not a rare condition. This was obviously for rare epilepsy, so even other people with epilepsy wouldn't necessarily fit in this group… So I think the online format was great, because it meant that those rare people could connect. And it was also good in that it just kind of fits in the schedule, too. It's an hour, it's not an hour plus travel time, and you can do it in your pyjamas at home*” [Carer 12].

Participants often missed sessions due to caring responsibilities and children’s bedtimes and appreciated the ability to access recordings of the retreat sessions that they missed. “*We still have access to all the things so I can still… go and look at a seminar again… You've got the resource there if you need it. And that's been good too, to have access to that after the fact. So I don't feel like I've wasted an opportunity*” [Carer 26]. The addition of a physical care package was praised as providing a personal touch that complemented the online delivery. “*It was really sweet having … those materials sent out in a …care hamper… It made it personalised given that it was a virtual retrea*t” [Carer 7].

### Program structure

The participants provided specific feedback on the structure of the program, what worked well and what could be further improved.

## Theme: “never going to be a perfect time” – varied experiences meant that no one offering suited everyone

The majority of the interviewed participants saw an hour per session as a good balance between the time that parents had available to attend sessions and the content that was able to be delivered. “*One of the best things for me was that each session was one hour, and that timeframe was respected. And within that time frame what they delivered was manageable. It wasn't overwhelming. We weren’t completing a PhD in fibre optics and physics, we were trying to build connections with people that are experiencing life being carers of children with rare epilepsy and in that situation there is an instant connection*” [Carer 7]. However, one participant indicated that the hour-long slot could have been extended to allow for the option of conversations to continue. *“Sometimes it got to the hour and it felt like people still had things to say, people were just getting going,…even having it flexible that it's an hour booked and then an optional half an hour to stay in it, so that if people need to leave after an hour they don't feel guilty about it, but it also leaves that window open for people to finish conversations”* [Carer 12].

One participant commented that they would have preferred a longer time period to allow for connections when people’s caregiving responsibilities meant they missed some sessions. “*I feel because it was quite rushed in length, six weeks, then it wasn't there. So I didn't make connections as I would have liked to with some of the people because sometimes I wasn’t there, and sometimes you wouldn't be there. So we sort of feel like we've kind of missed a lot of people*” [Carer 11].

There was disagreement on the feasibility of attending three evening retreat sessions per week, which may reflect the heterogeneity of experiences that come with caring for a child with a rare disease. Two different parents described it as a negative to have three sessions per week, because they found it difficult to attend every session. “*I'm caring full time and my husband works full time, so it was just hard to juggle*” [Carer 11]. But another parent framed this structure positively because it allowed them flexibility to make at least one session each week, and not feel like they were missing too much by missing one session. “*It's also nice having the three sessions because then if you only turn up to two, you're still getting something out of it. Whereas if there were only two sessions, and you missed one, then you're missing quite a lot*” [Carer 12]. Another “*would have preferred even just once a week, and for it to go for longer. Because in some sessions I felt like I was behind*” [Carer 11]. This disparity may be due to variable caring responsibilities and children’s bedtimes, making it impossible for a single time to suit every family. “*It's probably never going to be a perfect time that is picked… Because it's in the middle of sort of bedtime and coming out of dinner time and all that kind of thing*” [Carer 15].

Each interviewee had something positive to say about the structure of the retreat in general. All requested to be part of ongoing monthly sessions beyond the initial retreat. “*I think the monthly catch up would be really good. And that could have a theme to it to maybe, like yoga*” [Carer 11]. The size of the group was described as conducive to connection and sharing. One participant appreciated the less structured sessions for the opportunity to connect with others. “*The open discussion was really nice because it was mum led”* [Carer 15] while another praised the structure of the retreat as a whole. *“I think it was so freakin’ well done. It was manageable. You could see the start, you could see the middle, you could see the end. I just take my hat off to whoever designed this program, they made it manageable, and they respected the finish times”* [Carer7].

## Carer wellbeing, quality of life, valuing, and social inclusion

Positive changes in all wellbeing domains were reported from baseline to follow-up (Table 4). The Accomplishment domain showed the most positive change, with 9 carers reporting a positive change and 1 reporting no change. Similarly, overall wellbeing score improved for 9/10 carers.

**Table 4:** Comparison of participants' PERMA profiles in Q1 and Q2

Domain	Median at Q1 (range)	Median at Q2 (range)	Change from Q1 to Q2
Positive emotion	5.0 (2.7–7.0)	6.5 (5–8.3)	Positive change = 8 Negative change = 1 No change = 1
Engagement	6.5 (3.3–8.3)	7.0 (5.0–9.0)	Positive change = 8 Negative change = 1 No change = 1
Relationships	5.8 (3–8.7)	7.0 (2.0–9.0)	Positive change = 7 Negative change = 3 No change = 0
Meaning	6.3 (3.3–9.0)	7.5 (5.3–9.3)	Positive change = 7 Negative change = 2 No change = 1
Accomplishment	5.3 (3.7–7.3)	7.0 (5.3–8.3)	Positive change = 9 Negative change = 0 No change = 1
Overall well-being	6.0 (4.2–7.9)	7.0 (5.1–8.5)	Positive change = 9 Negative change = 1 No change = 0

The impact of the retreat on self-reported quality of life varied by domain, with the greatest number of participants reporting positive change in the Control over daily life and Self-care domains (Table 5). A small number of participants reported negative changes in quality of life, while most reported no change. In addition, the majority of participants reported positive change in the valuing indicators from baseline to follow-up (Table 6). The median score on the Progress domain increased, indicating a higher degree of valued living over time, while the lower score on the Obstruction domain indicates improvements in this domain.

**Table 5:** Changes in self-reported quality of life from Q1 (baseline) to Q2 (follow-up)

Domain/Item	Rating	Participants (n)		Change from Q1 to Q2
		Q1 (n = 10)	Q2 (n = 10)	
Domain 1: occupation (i.e., doing things one values or enjoys with their time)	Ideal state	0	0	Positive change = 1 Negative change = 1 No change = 8
	No needs	1	0	
	Some needs	8	10	
	High level needs	1	0	
Domain 2: control over daily life	Ideal state	0	1	Positive change = 5 Negative change = 1 No change = 4
	No needs	2	5	
	Some needs	7	3	
	High level needs	1	1	
Domain 3: self-care (i.e., look after oneself)	Ideal state	1	0	Positive change = 5 Negative change = 3 No change = 2
	No needs	1	2	
	Some needs	4	6	
	High level needs	4	2	
Domain 4: personal safety	Ideal state	8	9	Positive change = 2 Negative change = 1 No change = 7
	No needs	2	1	
	Some needs	0	0	
	High level needs	0	0	
Domain 5: social participation/contact	Ideal state	1	2	Positive change = 4 Negative change = 2 No change = 4
	No needs	1	1	
	Some needs	7	7	
	High level needs	1	0	
Domain 6: space and time to be yourself	Ideal state	1	0	Positive change = 4 Negative change = 2 No change = 4
	No needs	1	2	
	Some needs	5	7	
	High level needs	3	1	
Domain 7: feeling supported and encouraged	Ideal state	2	2	Positive change = 4 Negative change = 2 No change = 4
	No needs	2	5	
	Some needs	6	2	
	High level needs	0	1

**Table 6:** Comparison of participants’ Valuing indicators in Q1 and Q2

Domain	Q1 (n = 10)	Q2 (n = 10)	Change from Q1 to Q2
	Median (range)	Median (range)	
Progress	29.5 (20.0–42.0)	36.0 (27.0–43.0)	Positive change = 8 Negative change = 2 No change = 0
Obstruction	32.5 (8.0–39.0)	25.0 (14.0–37.0)	Positive change = 7 Negative change = 3 No change = 0

Overall, most participants reported positive changes in perceived social inclusion (Table 7). For all domains, the number of participants reporting positive change was greater than the number reporting negative change, with the greatest positive change being observed in the social relationships domain, indicating participants increased the amount of contact they had with people and society.

**Table 7:** Comparison of participants' Social Inclusion indicators in Q1 and Q2

Domain	Q1(n = 10)	Q2 (n = 10)	Change from Q1 to Q2
	Median (range)	Median (range)	
Social isolation	14.5 (6.0–17.0)	16.0 (10.0–18.0)	Positive Change = 6
			Negative change = 4
			No change = 0
Social relationships	20.0 (17.0–28.0)	23.5 (15.0–26.0)	Positive change = 7
			Negative change = 2
			No change = 1
Social acceptance	15.0 (11.0–19.0)	14.5 (10.0–19.0)	Positive change = 6
			Negative change = 4
			No change = 0

## Theme: “it's so difficult to put yourself first”

Thanks to the retreat, carers of children with rare epilepsy reported that they felt more empowered to put effort into their own needs and wellbeing.

“*I might not always execute it, but I'm definitely more mindful of trying to put myself first*” [Carer 26].

The participants interviewed also acknowledged that they usually would not take time for themselves and always prioritise caring for others over caring for themselves.

*“A lot of the time, us mums tend to put ourselves at the back, at the end. And it builds up to a point where it's like, there's no return. How do I come back from that? And then since you've been there for so long, fighting, fighting, fighting, it's like, well, what do I do? So having that opportunity to see other mums and how you can [come back] even in small ways, I think that opens up windows for you to say, okay, I can breathe”* [Carer 15]*.*

*“The importance of taking time when you can, even if it's just five minutes here and five minutes there. Because as a carer, or from my perspective anyway, but I think more generally as carers, it's so difficult to put yourself first”* [Carer 12].

They said the retreat encouraged them to focus on their own wellbeing and take opportunities to do activities that they find fun and fulfilling.

“*I was already trying to look at what I could sort of maybe outsource or do I quit my job, or whatever it is, something's got to give. Like, it can't all be everything. And I've been told if I like my job, keep it because it's the last one thing I have left for myself, even though it's working and not pleasure*” [Carer 26].

“*It's just opened me up to doing other things like maybe joining a meditation group or going to yoga, things like that, and realising those things. So yeah, I think it's sort of helped me refocus a bit on things I can do, really basic things to help myself at any time*” [Carer 9].

## Theme: “you operate in a parallel universe” – caregiving is isolating

The mothers in the interviews reflected on the social isolation that comes with caring for a child with rare epilepsy, including isolation from the wider community and from family members and partners.

“*It's a shame because we'd love to go and do things together. But we kind of split it up into separate things*” [Carer 11].

Participants reflected that the demands of caregiving leave little to no capacity for forming and nurturing relationships.

“*I'm just moving from hospital to hospital, hospital to medical appointments, specialists, Zoom teams, different settings, different medical specialties. I don't have time to attend a marriage, let alone friendships. My dog's not even living here because I don't have time to look after our dog. I know we all view our life through the prism of now, my prism of now is that I don't have time to look after other people and build new connections and look after those new connections*” [Carer 7].

The retreat was described as an opportunity to connect with other parents who were also experiencing and understood this isolation.

“*The whole experience of being in this space of a child with unique or rare or complex epilepsy is isolating in and of itself. You operate in a parallel universe. Being in that group, and groups like it, of people that are living similar experiences is reassuring and you feel like you have a space in the world where you're valued and seen*” [Carer 7].

“*For me, it was just a chance to connect with other people when I felt really alone. That was probably the main benefit I got out of it*” [Carer 9].

## Theme: “there’s no replacement for shared experience”

The group valued not having to explain themselves or their situations thanks to their shared experience.

“*It was just nice to have a little community of people that you didn't even have to go into detail of your story. They just knew. They understand. There's an understanding*” [Carer 7].

“*There isn't a replacement, I think, for that shared experience*” [Carer 12].

The retreat offered the rare opportunity for these carers to discuss their experiences amongst others who understood their challenges.

“*Just realising that I wasn't alone [and that] other people are living this journey was profound. So it's simply having the chance to sit in the same space as other people who I’d never meet any other way*” [Carer 9].

“*I can talk to my friends or I can talk to family or I can talk to my counsellor about stuff that's happening. But people can't comprehend actually what you're going through. And they just end up saying things like, ‘oh, I don't know how you cope’. It's not helpful for you. No, but nobody in that group said, ‘oh, I don't know how you cope’. Nobody made you feel different*” [Carer 12].

When comparing this retreat to another mental wellbeing retreat, one carer described it as more beneficial “*Because of the specific connection to epilepsy and that peer or lived experience where everyone just understands, and you don't even really have to say that much, people just understand people*” [Carer 12].

## Discussion

This pilot study evaluated the feasibility and acceptability of the Raregivers Wellness Retreat and assessed the preliminary impact of the retreat on participants’ wellbeing and social connectedness. The Wellness Retreat program, delivered virtually over 6 weeks, was judged to be feasible and highly acceptable by the 10 participants who completed pre-and post-intervention surveys. Carers reported improvements in several wellbeing domains measured by the PERMA Profiler, including positive emotion, engagement, relationships, meaning, and accomplishment. Furthermore, gains were observed in aspects of carer quality of life (measured by the ASCOT-SCT4), particularly in domains such as control over daily life and self-care. Similarly, positive changes in social inclusion were noted, with carers reporting greater social connection post-intervention.

Key identified strengths of the program were the flexible online nature of the Retreat combined with the authentic knowledge of the facilitators about the impact of the carer role, speaking to the critical role of co-design in models of care for rare disease [25, 26]. This framework enabled participants to learn practical skills that they could integrate into their busy lives and improve their social connectedness, which had the potential to have longer term positive impacts on their wellbeing.

The evaluation findings highlighted the profound impact of being a carer of a child with complex needs. This aligns with previous research indicating that carers of children with rare diseases experience substantial mental and emotional challenges, often compounded by isolation and a lack of targeted support systems [4, 10–12]. Studies such as those by Reilly et al. (2018) [6] and Jakobsen et al. (2020) [5] have demonstrated the heightened risk of depression, anxiety, and stress among carers of children with epilepsy, and that having a child with a rare epilepsy has a particularly high impact on wellbeing, with carers being in a state of chronic traumatic stress. Our results confirm the benefits of structured wellness programs in mitigating these challenges and provide evidence of the feasibility and strengths of such programs. These programs have the potential advantages of connecting carers who, due to the rarity of their child’s condition, are often geographically distant from others with similar lived experience [13]. The findings highlight the importance of social inclusion in the caregiving experience, with improvements in social connectedness reported by participants suggesting that creating structured peer support networks can mitigate feelings of isolation, which is a key contributor to poor mental health in people living with rare disease [2, 27].

Importantly, this study contributes new insights into the feasibility of adapting programs developed in other countries to the Australian context, where there is a shortage of evidence-based wellness interventions for rare disease carers and acknowledged scarcity of publicly funded mental health and wellbeing services [10, 13, 25].

Positive psychology interventions have been used successfully in carer populations [28, 29], but their application in rare disease caregiving is less explored. Our findings suggest that the Raregivers Wellness Retreat may be particularly sensitive to promoting improvements in carers' overall wellbeing by fostering positive emotions, meaning, and accomplishment—critical components in sustaining resilience in long-term caregiving roles.

## Implications for future research and practice

The findings from this study offer insights for future research and the design of support programs for carers of children with rare diseases. First, the high levels of program satisfaction and reported improvements in wellbeing support the integration of carer wellness programs into rare disease health care frameworks. Further, these findings show that structured, well-designed programs like the Raregivers Wellness Retreat can make a meaningful difference in the lives of carers. The success of the online format and peer group setting suggests these components could be integral to future interventions, offering the flexibility and community support that are so crucial for this group [30]. Given the chronic, complex nature of rare epilepsy syndromes and the intense caregiving demands, targeted programs like the Raregivers Wellness Retreat could be invaluable in alleviating carer stress and improving quality of life. Expansion of the programme to other conditions would be potentially beneficial.

Given the promising results, future research should expand the scope of the program by recruiting larger cohorts and incorporating control groups to strengthen the evaluation of its effectiveness. There is a strong need for robust research to support the health and wellbeing of carers as part of the delivery of integrated health care [31]. Moreover, longitudinal studies would be valuable in assessing the long-term impact of wellness interventions on carer wellbeing and whether the positive changes observed can be sustained over time.

This study's strengths include its mixed-methods design, which enabled a comprehensive evaluation of the program’s impact on carers' wellbeing through both quantitative and qualitative data. In addition, the program was co-designed by parents of children with rare epilepsy and adapted for the Australian context.

However, the study is not without limitations. The small sample size, while understandable for a pilot study, limits the generalisability of the findings. Although the program was widely advertised on social media and through clinical networks, most individuals attending were well-educated mothers who did not identify as being from a First Nations background and or a culturally or linguistically diverse community. Careful co-design with priority populations will be required to improve the diversity and cultural appropriateness of programs, which require appropriate resourcing, time and leadership from diverse communities [32, 33].

Future studies with larger and more diverse cohorts of participants are needed to confirm these results. Additionally, as a pilot, the absence of a control group prevents definitive conclusions about the program’s effectiveness. Self-reported measures, though useful for capturing subjective experiences, may introduce bias, particularly in post-intervention assessments where participants may feel inclined to report positive outcomes. Moreover, as a pilot, the study was not powered to test dose–response (‘intervention dose’) effects; while attendance varied, the small sample and individual variability precluded meaningful inference. Future trials should pre-specify minimum exposure, capture broader engagement (e.g., recordings, home practice), and evaluate dose–response.

We acknowledge that child clinical characteristics likely influence caregiver burden and program response. Given the pilot design and limited sample, we did not analyse or report associations between child-level factors and outcomes. This choice prioritised establishing acceptability and safety but limits inferences about independent effects of child characteristics and may constrain generalisability. Future research should be powered to include prespecified child-level covariates (e.g., severity/duration markers, age, functional dependence) in multivariable or mixed-effects models to quantify their contribution to caregiver outcomes.

Excluding individuals with high distress scores may have introduced selection bias, as carers with more acute psychological needs—who arguably have the greatest need for intervention—were not represented in this evaluation. While necessary on ethical and safety grounds for a pilot focused on establishing feasibility and acceptability, this may mean findings reflect carers with moderate levels of stress/distress and may limit generalisability. Future iterations should test more inclusive models (e.g., stepped-care approaches), integrating screening and clear referral pathways so that participants with higher distress can access parallel clinical supports while participating, thereby preserving psychological safety and broadening applicability.

There is also potential to explore the application of similar wellness programs across different carer populations, particularly those caring for children with other complex rare diseases. The success of the virtual format suggests that such programs could be effectively scaled and tailored to meet the needs of different communities.

## Conclusions

The Raregivers Wellness Retreat was feasible and acceptable, and may potentially have a positive impact on the wellbeing, quality of life, and social connectedness of carers of children with rare epilepsies. The program’s high acceptability, coupled with positive changes in wellbeing and quality of life, provides a strong foundation for further development and implementation. These findings support the broader adoption of structured wellness programs in rare disease care, with the potential to improve the lives of carers who face considerable emotional, physical, and social burdens.

## Supplementary Information

Additional file1

Additional file2

Additional file3

Additional file4

## Data Availability

De-identified datasets used and/or analysed during the current study are available from the corresponding author on reasonable request.

## Abbreviations

ARIA: Accessibility/Remoteness Index of Australia Plus
ASCOT-SCT4: Adult Social Care Outcomes Toolkit - Self-completion Tool 4
DEE: Developmental and Epileptic Encephalopathy
GETA: Genetic Epilepsy Team Australia
IRSD: Index of Relative Socio-Economic Disadvantage
PERMA: Positive emotion, Engagement, Relationships, Meaning, and Accomplishment
REDCap: Research Electronic Data Capture
SEIFA: Socio-Economic Indexes for Areas
SES: Socio-Economic Status
SIS: Social Inclusion Scale
UNSW: University of New South Wales
