# Supplementary material for: “Just realising that I wasn’t alone… was profound”: a mixed-methods evaluation of a pilot peer-to-peer wellbeing program for carers of children with rare epilepsies
Source: Orphanet J Rare Dis. 2025 Oct 21;20:524. doi: 10.1186/s13023-025-04036-0 (PMC12538871; doi:10.1186/s13023-025-04036-0)
Supplement: Supplementary file 1 — Additional file1 [file 13023_2025_4036_MOESM1_ESM.pdf]

Raregivers Wellness Retreat: Screening Questionnaire

Thank you for your interest in the Wellness Retreat.

To make sure that this program is suitable for you, we ask that you complete this short, 5 minute questionnaire:

	**Question**	**Response**
**1**	What is your full name?	*Open*
**2**	What is your preferred email address?	*Open*
**3**	What is your mobile number?	*Open*
**4**	What is your relationship to the child that you care for?	*Open*
**5**	Does your child's rare genetic epilepsy have a name? If so, please list it here	*Open*

6. **a)** First please circle the number 0-10 that best describes how much distress you have been experiencing in the past week including today.

b) Second, please indicate if any of the following has been a problem for you in the past week including today. Be sure to check YES or No for each.

First please circle the number (0-10) that best describes how much distress you have been experiencing in the past week including today.

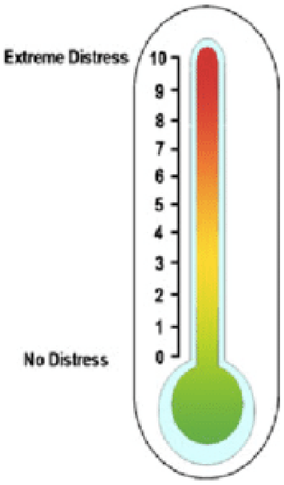

Second, please indicate if any of the following has been a problem for you in the past week including today. Be sure to check YES or NO for each.

YES NO Practical Problems
- ☐ ☐ Child Care
- ☐ ☐ Housing
- ☐ ☐ Insurance/financial
- ☐ ☐ Transportation
- ☐ ☐ Work/school

Family Problems
- ☐ ☐ Dealing with children
- ☐ ☐ Dealing with partner
- ☐ ☐ Dealing with close Friend/relative

Emotional Problems
- ☐ ☐ Depression
- ☐ ☐ Fears
- ☐ ☐ Nervousness
- ☐ ☐ Sadness
- ☐ ☐ Worry
- ☐ ☐ Loss of interest in usual activities

- ☐ ☐ Spiritual/religious concerns

YES NO Physical Problems
- ☐ ☐ Appearance
- ☐ ☐ Bathing/dressing
- ☐ ☐ Breathing
- ☐ ☐ Changes in urination
- ☐ ☐ Constipation
- ☐ ☐ Diarrhoea
- ☐ ☐ Eating
- ☐ ☐ Fatigue
- ☐ ☐ Feeling Swollen
- ☐ ☐ Fevers
- ☐ ☐ Getting around
- ☐ ☐ Indigestion
- ☐ ☐ Memory/concentration
- ☐ ☐ Mouth sores
- ☐ ☐ Nausea
- ☐ ☐ Nose dry/congested
- ☐ ☐ Pain
- ☐ ☐ Sexual
- ☐ ☐ Skin dry itchy
- ☐ ☐ Sleep
- ☐ ☐ Tingling in hands/feet

Other problems

____

____
