# Supplementary material for: “Just realising that I wasn’t alone… was profound”: a mixed-methods evaluation of a pilot peer-to-peer wellbeing program for carers of children with rare epilepsies
Source: Orphanet J Rare Dis. 2025 Oct 21;20:524. doi: 10.1186/s13023-025-04036-0 (PMC12538871; doi:10.1186/s13023-025-04036-0)
Supplement: Supplementary file 3 — Additional file3 [file 13023_2025_4036_MOESM3_ESM.pdf]

Raregivers Wellness Retreat: Follow-up Questionnaire

Thank you for completing this follow-up questionnaire for the Wellness Retreat evaluation. Some of the questions may seem a little bit repetitive. However, every question we ask is important for our research study. This will make sure that this study is meaningful and can be used to help other Caregivers.

Section 1: Your Feedback about the Program

How would you rate the quality of the service you received with the Wellness Retreat?

◯ Excellent
◯ Good
◯ Fair
◯ Poor

To what extent has the Wellness Retreat met your needs?

◯ Almost all of my needs have been met
◯ Most of my needs have been met
◯ Only a few of my needs have been met
◯ None of my needs have been met

If a friend were in need of similar help, would you recommend the Wellness Retreat to them?

◯ Yes, definitely
◯ Yes, generally,
◯ No, not really
◯ No, definitely not

The Program length should have been

◯ Less than 6 weeks
◯ It was the right length
◯ More than 6 weeks

The workshop sessions were

◯ Too short
◯ They were the right length
◯ Too long

The Mighty Networks online platform was easy to use

◯ Strongly Agree
◯ Somewhat Agree
◯ Somewhat Disagree
◯ Strongly Disagree

The online format of the retreat suited me

◯ Strongly Agree
◯ Somewhat Agree
◯ Somewhat Disagree
◯ Strongly Disagree

I think the online platform is user friendly

◯ Strongly Agree
◯ Somewhat Agree
◯ Somewhat Disagree
◯ Strongly Disagree

I had enough time and energy to complete the activities and workshops

◯ Strongly Agree
◯ Somewhat Agree
◯ Somewhat Disagree
◯ Strongly Disagree

The Sustainable Caregiver workshops were relevant to my experience

◯ Strongly Agree
◯ Somewhat Agree
◯ Somewhat Disagree
◯ Strongly Disagree

The workshops on Sensory Self-Care were relevant to my experience	◯ Strongly Agree<br>◯ Somewhat Agree<br>◯ Somewhat Disagree<br>◯ Strongly Disagree
I enjoyed/found it beneficial having other people in the group	◯ Strongly Agree<br>◯ Somewhat Agree<br>◯ Somewhat Disagree<br>◯ Strongly Disagree
I formed meaningful connections with other Caregivers during this retreat	◯ Yes<br>◯ Somewhat<br>◯ No
As a result of the Wellness Retreat, I have incorporated self-care and wellness practices into my own time and routines	◯ Yes<br>◯ Somewhat<br>◯ No
The practices learned have translated to me feeling less stressed day to day	◯ Yes<br>◯ Somewhat<br>◯ No

Caregiver Satisfaction Survey

Instructions: For the following questions, please choose the statement that best corresponds to your views about the program.

	Yes	No	Somewhat
Did you like the help you were getting?	○	○	○
Did you get the help you wanted?	○	○	○
Did you need more help than you got?	○	○	○
Were you given more services than you needed?	○	○	○
Have the services helped you with your life?	○	○	○

Section 2: Your General Wellbeing

Instructions: For the following questions, please choose the number that best corresponds to your views.

How much of the time do you feel you are making progress towards accomplishing your goals?

0 (Never) 10 (Always)

(Place a mark on the scale above)

How often do you become absorbed in what you are doing?

0 (Never) 10 (Always)

(Place a mark on the scale above)

In general, how often do you feel joyful?

0 (Never) 10 (Always)

(Place a mark on the scale above)

How often do you achieve the important goals you have set for yourself?

0 (Never) 10 (Always)

(Place a mark on the scale above)

In general, to what extent do you lead a purposeful and meaningful life?

0 (Not at all) 10 (Completely)

(Place a mark on the scale above)

To what extent do you receive help and support from others when you need it?

0 (Not at all) 10 (Completely)

(Place a mark on the scale above)

In general, to what extent do you feel that what you do in your life is valuable and worthwhile?

0 (Not at all) 10 (Completely)

(Place a mark on the scale above)

In general, to what extent do you feel excited and interested in things?

0 (Not at all) 10 (Completely)

(Place a mark on the scale above)

In general, how often do you feel positive?

0 (Never) 10 (Always)

(Place a mark on the scale above)

How often are you able to handle your responsibilities?

0 (Never) 10 (Always)

(Place a mark on the scale above)

How often do you lose track of time while doing something you enjoy?

0 (Never) 10 (Always)

(Place a mark on the scale above)

To what extent do you feel loved?	0 (Not at all)	10 (Completely)
	(Place a mark on the scale above)	
To what extent do you generally feel you have a sense of direction in your life?	0 (Not at all)	10 (Completely)
	(Place a mark on the scale above)	
How satisfied are you with your personal relationships?	0 (Not at all)	10 (Completely)
	(Place a mark on the scale above)	
To what extent do you feel contented?	0 (Not at all)	10 (Completely)
	(Place a mark on the scale above)	

Section 3: Social Relationships with Others

Instructions: For the following questions, please choose the statement that best corresponds to your views during the past month, including today.

	Not at all	Not particularly	Yes, a bit	Yes, definitely
I have felt terribly alone and isolated	◯	◯	◯	◯
I have felt accepted by my friends	◯	◯	◯	◯
I have been out socially with friends	◯	◯	◯	◯
I have felt I am playing a useful part in society	◯	◯	◯	◯
I have friends I see or talk to every week	◯	◯	◯	◯
I have felt what I do is valued by others	◯	◯	◯	◯
I have been to new places	◯	◯	◯	◯
I have learnt something about other cultures	◯	◯	◯	◯
I have been involved in a group not just for my university studies	◯	◯	◯	◯
I have done some cultural activity	◯	◯	◯	◯
I have felt some people look down on me because of how I am	◯	◯	◯	◯
I have felt unsafe to walk alone in my neighbourhood in daylight	◯	◯	◯	◯
I have friends I see or talk to every week	◯	◯	◯	◯
I have felt accepted by my neighbours	◯	◯	◯	◯
I have felt accepted by my family	◯	◯	◯	◯
I have felt clear about my rights I have	◯	◯	◯	◯
I have felt free to express my beliefs	◯	◯	◯	◯

Section 4: Living with your Values Instructions: Please read each statement carefully and then choose the number which best describes how much the statement was true for you during the past week, including today.

I spent a lot of time thinking about the past or future, rather than being engaged in activities that mattered to me	0 (Not true at all) — 10 (Completely true) *(Place a mark on the scale above)*
I was basically on "auto-pilot" most of the time	0 (Not true at all) — 10 (Completely true) *(Place a mark on the scale above)*
I worked toward my goals even if I didn't feel motivated to	0 (Not true at all) — 10 (Completely true) *(Place a mark on the scale above)*
I was proud about how I lived my life	0 (Not true at all) — 10 (Completely true) *(Place a mark on the scale above)*
I made progress in the areas of my life I care most about	0 (Not true at all) — 10 (Completely true) *(Place a mark on the scale above)*
Difficult thoughts, feelings or memories got in the way of what I really wanted to do	0 (Not true at all) — 10 (Completely true) *(Place a mark on the scale above)*
I continued to get better at being the kind of person I wanted to be	0 (Not true at all) — 10 (Completely true) *(Place a mark on the scale above)*
When things didn't go according to plan, I gave up easily	0 (Not true at all) — 10 (Completely true) *(Place a mark on the scale above)*
I felt like I had a purpose in life	0 (Not true at all) — 10 (Completely true) *(Place a mark on the scale above)*
It seemed like I was just 'going through the motions,' rather than focusing on what was important to me	0 (Not true at all) — 10 (Completely true) *(Place a mark on the scale above)*

Section 5: Your Quality of Life Instructions: Please read each statement carefully and then choose the number which best describes how much the statement was true for you during the past month, including today.

Which of the following statements best describes how you spend your time? (please select one box)

When you are thinking about how you spend your time, please include anything you value or enjoy, including leisure activities, formal employment, voluntary or unpaid work, and caring for others.

◯ I'm able to spend my time as I want, doing things I value or enjoy
◯ I'm able to do enough of the things I value or enjoy with my time
◯ I do some of the things I value or enjoy with my time, but not enough
◯ I don't do anything I value or enjoy with my time

Which of the following statements best describes how much control you have over your daily life? (please select one box)

◯ I have as much control over my daily life as I want
◯ I have adequate control over my daily life
◯ I have some control over my daily life, but not enough
◯ I have no control over my daily life

Thinking about how well you look after yourself - such as, getting enough sleep or eating well - which statement best describes your present situation?

◯ I look after myself as well as I want
◯ I look after myself well enough
◯ Sometimes I can't look after myself well enough
◯ I feel I am neglecting myself

Which of the following statements best describes how safe you feel?

By 'feeling safe' we mean feeling safe from fear of abuse, being attacked or other physical harm, such as accidents, which are a result of your caring role.

◯ I feel as safe as I want
◯ Generally I feel adequately safe, but not as safe as I would like
◯ I feel less than adequately safe
◯ I don't feel at all safe

Thinking about how much contact you have with people you like, which of the following statements best describes your social situation?

◯ I have as much social contact as I want with people I like
◯ I have adequate social contact with people
◯ I have some social contact with people, but not enough
◯ I have little social contact with people and feel socially isolated

Thinking about the space and time you have to be yourself in your daily life, which of the following statements best describes your present situation?

◯ I have all the space and time I need to be myself
◯ I have adequate space and time to be myself
◯ I have some of the space and time I need to be myself, but not enough
◯ I don't have any space or time to be myself

Thinking about feeling supported and encouraged in your caring role, which of the following statements best describes your present situation?

This question is asking about feeling supported and encouraged, rather than how you are supported and encouraged by particular people or organisations.

◯ I feel I have the encouragement and support I want
◯ I feel I have adequate encouragement and support
◯ I feel I have some encouragement and support, but not enough
◯ I feel I have no encouragement and support

Final Questions

Would you be willing to participate in a 30 minute interview about your experiences with the Retreat Program?	◯ Yes ◯ No
What is your best contact number?	____________________
When is the best day/time to call you? We will first call to answer any questions you may have and to book in your interview.	________________________
