# Supplementary material for: “Just realising that I wasn’t alone… was profound”: a mixed-methods evaluation of a pilot peer-to-peer wellbeing program for carers of children with rare epilepsies
Source: Orphanet J Rare Dis. 2025 Oct 21;20:524. doi: 10.1186/s13023-025-04036-0 (PMC12538871; doi:10.1186/s13023-025-04036-0)
Supplement: Supplementary file 4 — Additional file4 [file 13023_2025_4036_MOESM4_ESM.pdf]

Raregivers Virtual Wellness Retreat: Interview Guide

Introduction

The moderator explains the objective of the interview. *These questions will help us to learn about your experiences with wellness retreat. It will also help us to understand how the program may impact your self-efficacy and social connectedness*.

Consent

Confirm consent and re-iterate that *you do not have to answer any question you do not want to and if any questions make you feel uncomfortable you can stop any time you like. The interview will be audio recorded, transcripts will be de-identified and any quotes used will not be attributed by name*.

General

1. What did you think of the wellness retreat?
2. What do you like best about the wellness retreat?
3. What types of activities did you do with your mentor during the program?
4. Did you experience any difficulties or challenges during the retreat?
5. How would you explain the importance of self-care as a caregiver?

Self-efficacy

6. What have you learnt during the retreat?
7. Have you improved skills in any areas? Can you give an example?
8. Have you developed new skills in any areas? Can you give an example?
9. How has the retreat affected your confidence in caring for yourself?
10. Do you feel you have made connections with other group members?
11. How might any new skills or learnings be useful in the future?
12. Can you think of any other ways in which program has positively affected your life?
13. Before the Caregiver Wellness Retreat, how supported did you generally feel?
14. After participating in the Caregiver Wellness Program, how supported do you generally feel?

Facilitators and Barriers

15. What are some barriers to participating in the program that you faced? In your experience, what would've been the best way to overcome these?
16. In what ways could the program be more inclusive?
17. What do you think were the benefits of doing the retreat in a group setting?
18. What parts of the program did you find most challenging? Why?

Sustainability

19. Do you think what you have learnt is sustainable for you? Please explain
20. Are you going to maintain contact with other group members?
21. If you had unlimited time and money, how do you see this being rolled out in Australia

Closing Question

22. Are there any other benefits to participating in the program that we have not discussed?
